# Mu Opioid Receptor Actions in the Lateral Habenula

**DOI:** 10.1371/journal.pone.0159097

**Published:** 2016-07-18

**Authors:** Elyssa B. Margolis, Howard L. Fields

**Affiliations:** Department of Neurology, The Wheeler Center for the Neurobiology of Addiction, Alcoholism and Addiction Research Group, University of California San Francisco, San Francisco, California, United States of America; University of Kentucky Medical Center, UNITED STATES

## Abstract

Increased activity of lateral habenula (LHb) neurons is correlated with aversive states including pain, opioid abstinence, rodent models of depression, and failure to receive a predicted reward. Agonists at the mu opioid receptor (MOR) are among the most powerful rewarding and pain relieving drugs. Injection of the MOR agonist morphine directly into the habenula produces analgesia, raising the possibility that MOR acts locally within the LHb. Consequently, we examined the synaptic actions of MOR agonists in the LHb using whole cell patch clamp recording. We found that the MOR selective agonist DAMGO inhibits a subset of LHb neurons both directly and by inhibiting glutamate release onto these cells. Paradoxically, DAMGO also presynaptically inhibited GABA release onto most LHb neurons. The behavioral effect of MOR activation will thus depend upon both the level of intrinsic neuronal activity in the LHb and the balance of activity in glutamate and GABA inputs to different LHb neuronal populations.

## Introduction

Drugs acting at the mu opioid receptor (MOR) are currently the most powerful and broadly effective analgesics. Their effectiveness is due in part to the wide distribution of MOR in circuits that transmit and modulate the nociceptive message. MOR agonists inhibit the terminals of primary afferent nociceptors [1], nociceptive spinal dorsal horn neurons [1, 2], and nociceptive transmission in the anterior cingulate cortex [3]. MOR agonists also produce analgesia by activating pain inhibiting neurons in the periaqueductal gray and rostral ventromedial medulla [4].

In addition to these canonical pain transmission and modulatory circuits, there is accumulating evidence for another significant brain circuit (or circuits) relaying through the lateral habenula (LHb) that generates aversive states, including contributing to the processing of nociceptive signals. The LHb is a major component of the epithalamus located the caudal dorsal thalamic region. Its component neurons are primarily glutamatergic, fire spontaneously *ex vivo* and are interconnected within the LHb [5, 6]. The LHb receives inputs from several forebrain and diencephalic structures. Subcortical input structures include the lateral hypothalamus, entopeduncular nucleus (EPN), ventral pallidum, ventral tegmental area (VTA), and nucleus accumbens [7]. The cortical afferents arise from the anterior cingulate and anterior insular cortex both of which have pain responsive neurons [8, 9]. Different LHb populations project directly to dopaminergic VTA or serotonergic (dorsal raphe nucleus (DRN)) neurons [10]. A third population of LHb output neurons excite GABAergic neurons in the caudal midbrain (the rostromedial tegmental nucleus (RMTg)) [11] and optogenetically stimulating the LHb input to RMTg produces aversion [12]. Separate subpopulations of RMTg neurons inhibit VTA dopamine and DRN serotonergic neurons [13]. Importantly, LHb neurons also project to the canonical pain modulating nucleus the periaqueductal gray [14, 15].

Consistent with a role in aversive signaling, LHb neuronal firing patterns encode a variety of negative outcomes and aversive states including pain and rodent models of depression. LHb neurons fire in response to aversive stimuli including tail pinch [16], sciatic nerve stimulation [17] and air puff to the face [18]. Optogenetic activation of glutamatergic inputs from the VTA to LHb neurons is also aversive [19], suggesting that increased activity of LHb neurons is sufficient to generate a negative state. Lesions of the habenula can block the analgesic effect of electrical stimulation in the lateral hypothalamus [20], indicating that it can be a critical node in a pain relief circuit.

The prominent role of LHb in aversive signaling and its connection with MOR sensitive brain regions implicated in both pain transmission and modulation raises the question of whether MOR agonists can act directly within the LHb to alleviate aversive states such as ongoing pain. Consistent with this idea, the MOR agonist morphine given systemically prevents the increase in LHb neural activity caused by pain [17] and morphine injection directly into the habenula can produce analgesia [21]. Morphine withdrawal induces fos activation in the LHb [22], which may reflect termination of MOR inhibition of LHb neurons. This action could contribute to the aversiveness of morphine abstinence. Here we examined pre- and post-synaptic elements in the LHb for sensitivity to MOR activation using whole cell patch clamp recordings in adult male rats. We hypothesized that since many LHb neurons are excited by pain, MOR activation should inhibit a subset of LHb neurons. We report that in fact the MOR selective agonist DAMGO directly hyperpolarizes a subset of LHb neurons and inhibits glutamate release on to that same subpopulation. GABA release was also partially inhibited by MOR activation. These synaptic actions provide a basis for understanding the behavioral effects of MOR agonists acting in the LHb.

## Materials and Methods

All animal protocols were conducted in strict accordance with the recommendations of the National Institutes Health (NIH) in the Guide for the Care and Use of Laboratory Animals. Research protocols were approved by the Institutional Animal Care and Use Committee (University of California at San Francisco, CA), approval ID AN091813-03D. Decapitation was performed following deeply anesthetizing the animals with isoflurane to minimize discomfort.

### Slice Preparation and Electrophysiology

Recordings were made in adult male Sprague-Dawley rats (>200 g). Rats were deeply anesthetized with isoflurane and then decapitated. Coronal brain slices (200 μm thick) were prepared using a vibratome (Leica Instruments). Slices were prepared in ice cold Ringer solution (in mM: 119 NaCl, 2.5 KCl, 1.3 MgSO_4_, 1.0 NaH_2_PO_4_, 2.5 CaCl_2_, 26.2 NaHCO_3_, and 11 glucose saturated with 95% O_2_-5% CO_2_) and allowed to recover at 33°C for at least 1 hr. Slices were visualized under a *Zeiss Axioskop FS 2 plus* with differential interference contrast optics and infrared illumination or an *Axio Examiner A1* also equipped with Dodt optics, each using a *Zeiss Axiocam MRm* and *Axiovision 4* (Zeiss) or *Microlucida* (MBF Biosciences) software. Whole cell recordings were made at 33°C using 4–5 MΩ pipettes. Recordings were made using *Axopatch 1-D* amplifiers (Axon Instruments), filtered at 5 kHz and collected at 20 kHz using *IGOR Pro* (Wavemetrics). For all current clamp experiments I = 0, for voltage clamp experiments V_m_ = -60 or -70 mV. For synaptic experiments, bipolar stimulating electrodes were placed in the LHb, and paired electrical pulses (50 ms interval) were delivered once every 10 s. Liquid junction potentials were not corrected during recordings. Input and series resistances were monitored with hyperpolarizing pulses (0.1 Hz) throughout each experiment.

When measuring glutamatergic synaptic events and for current clamp experiments, the pipette solution contained (in mM): 123 K-gluconate, 10 HEPES, 0.2 EGTA, 8 NaCl, 2 MgATP, 0.3 Na_3_GTP, and 0.1% biocytin (pH 7.2, osmolarity adjusted to 275 mOsm/L). To measure glutamatergic synaptic events recordings were completed in the presence of 20 μM gabazine to block GABA_A_R mediated synaptic events. GABA_A_R mediated synaptic events were recorded in the presence of 10 μM DNQX using an internal solution containing (in mM): 128 KCl, 20 NaCl, 1 MgCl_2_, 1 EGTA, 0.3 CaCl_2_, 10 HEPES, 2 MgATP and 0.3 Na_3_GTP (pH 7.2, osmolarity adjusted to 275 mOsm/L). All drugs were applied by bath perfusion.

Recordings were completed throughout the anterior-posterior extent of the LHb, mostly within either the central part of the medial division of the LHb or the adjacent magnocellular part of the lateral division of the LHb [23]. No relationships were observed between MOR actions and recording location.

Agonists, antagonists, salts, ATP, and GTP were obtained from MilliporeSigma or Tocris (Bio-Techne).

### Data Analysis

Results are presented as mean ± S.E.M. Drug effects were statistically evaluated in each neuron by binning data into 30 s data points and comparing the last 8 baseline data points to the last 8 data points during drug application using Student’s unpaired *t* test. All neurons recorded in current clamp were quiescent during DAMGO testing, therefore membrane potential was analyzed in all cases. To measure the amplitude of electrically evoked postsynaptic currents, a 2 ms period just before the stimulation artifact was compared to a 2 ms period around the peak current. The paired pulse ratio (PPR) was calculated by dividing the second evoked amplitude by the first. Spontaneous and miniature postsynaptic currents were identified by searching the smoothed first derivative of the data trace for values that exceeded a set threshold, and these events were visually confirmed. Experiments where the event frequency was < 0.5 Hz were not included in the analysis because too few events were measured for reliable quantification. Statistical comparisons between groups of neurons were made using one way ANOVAs. Other statistical tests are indicated in the text where they were applied. *p* < 0.05 was required for significance in all analyses.

## Results

**Fig 1:**
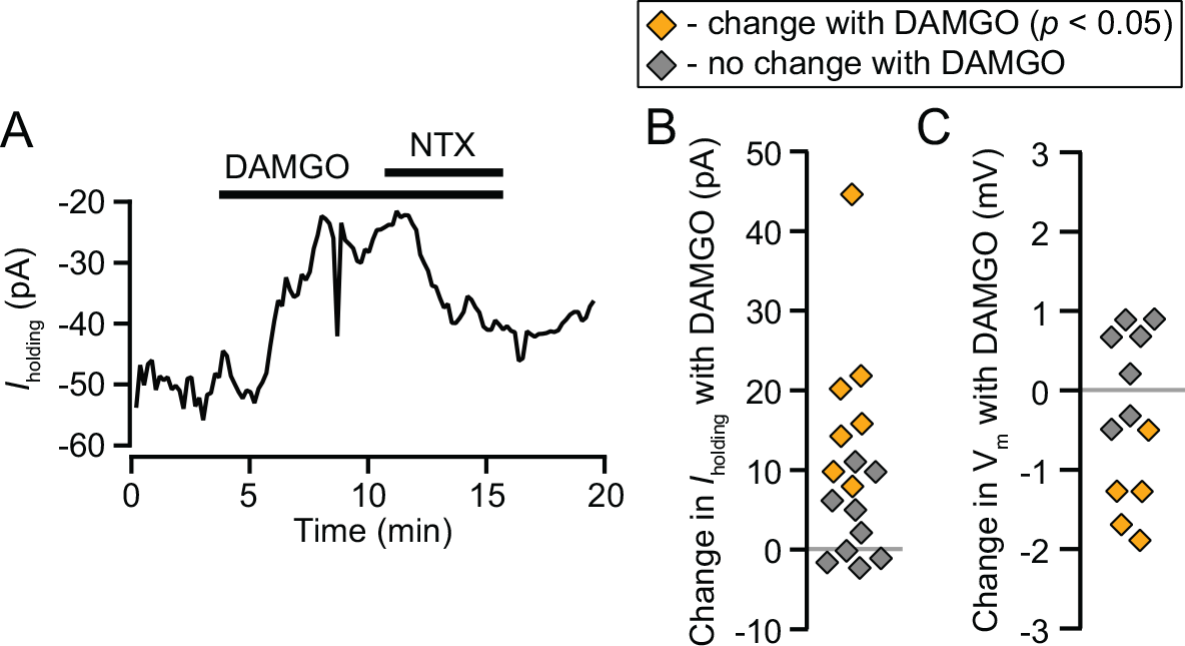
MOR activation hyperpolarizes a subset of LHb neurons. (A) Example voltage clamp recording of an LHb neuron where DAMGO (500 nM) produced an outward current, reversed by naltrexone (1 μM). (B) 500 nM DAMGO induced a significant outward current in 7 out of 15 neurons. (C) In current clamp (I = 0 pA), 5 out of 12 neurons were significantly hyperpolarized by 500 nM DAMGO.

We hypothesized that MOR activation in the LHb would decrease its output activity. The most direct synaptic action for this effect is a postsynaptic hyperpolarization. In voltage clamp experiments (V_m_ = -60 or -70 mV) we tested LHb neurons for changes in holding current during bath application of the MOR selective agonist DAMGO (500 nM). DAMGO caused a small but significant outward current in a subset of neurons (7/15; 16.8 ± 5.0 pA; Fig 1A and 1B). Outward currents were partially reversed by the MOR selective antagonist CTAP (n = 4; 57 ± 11% recovery to baseline). In current clamp experiments (I = 0), we observed a hyperpolarization in response to DAMGO in a subset (5/12) of LHB neurons (Fig 1C). Among all 12 DAMGO sensitive neurons (current and voltage clamp experiments), increases in conductance were also observed in 6 neurons, suggesting a channel opening. No depolarizations or inward currents were observed in response to DAMGO.

**Fig 2:**
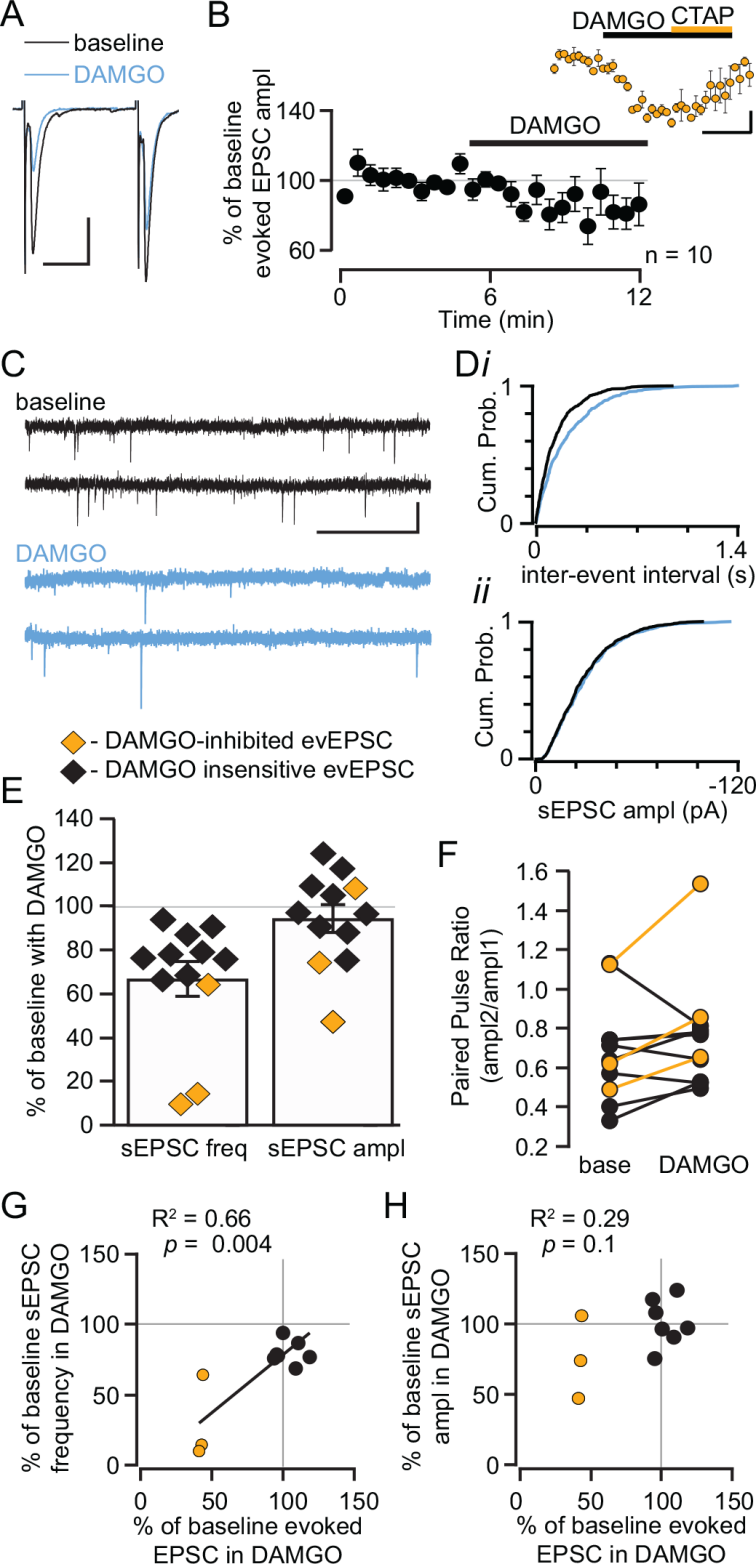
MOR activation inhibits glutamate release onto a subset of LHb neurons. (A) Example traces of glutamate mediated evoked EPSCs in a cell showing an inhibition in response to 500 nM DAMGO. Scale bars 100 pA and 20 ms. (B) Summary of evoked EPSC time course across all 10 neurons tested with DAMGO. Inset: In the 3 neurons where DAMGO caused a significant decrease in the amplitude of the evoked EPSCs, the MOR selective antagonist CTAP reversed this effect. Scale bars 25% and 5 min. (C) Example traces from a neuron where DAMGO decreased the frequency, but not the amplitude, of sEPSCs. Scale bars 40 pA and 50 ms. The cumulative probability plots for this same neuron (D) show a shift to larger inter-event intervals (*i*) indicating a decrease in event frequency, but no change in event amplitude (*ii*), during DAMGO application. Across all neurons where sEPSCs were measured, the frequency of sEPSCs decreased to at least some extent during DAMGO application (E, left). There was no consistent change in sEPSC amplitude in response to DAMGO (E, right). (F) In the 3 neurons where DAMGO inhibited the amplitude of the evoked EPSCs, the PPR also increased. (G) Across neurons where both electrically evoked EPSCs and sEPSCs were measured, there was a significant direct correlation between the inhibition of the amplitude of evoked EPSCs by DAMGO and the inhibition of sEPSC frequency. (H) There was no relationship between the effect of DAMGO on evoked and spontaneous EPSC amplitude across neurons.

**Fig 3:**
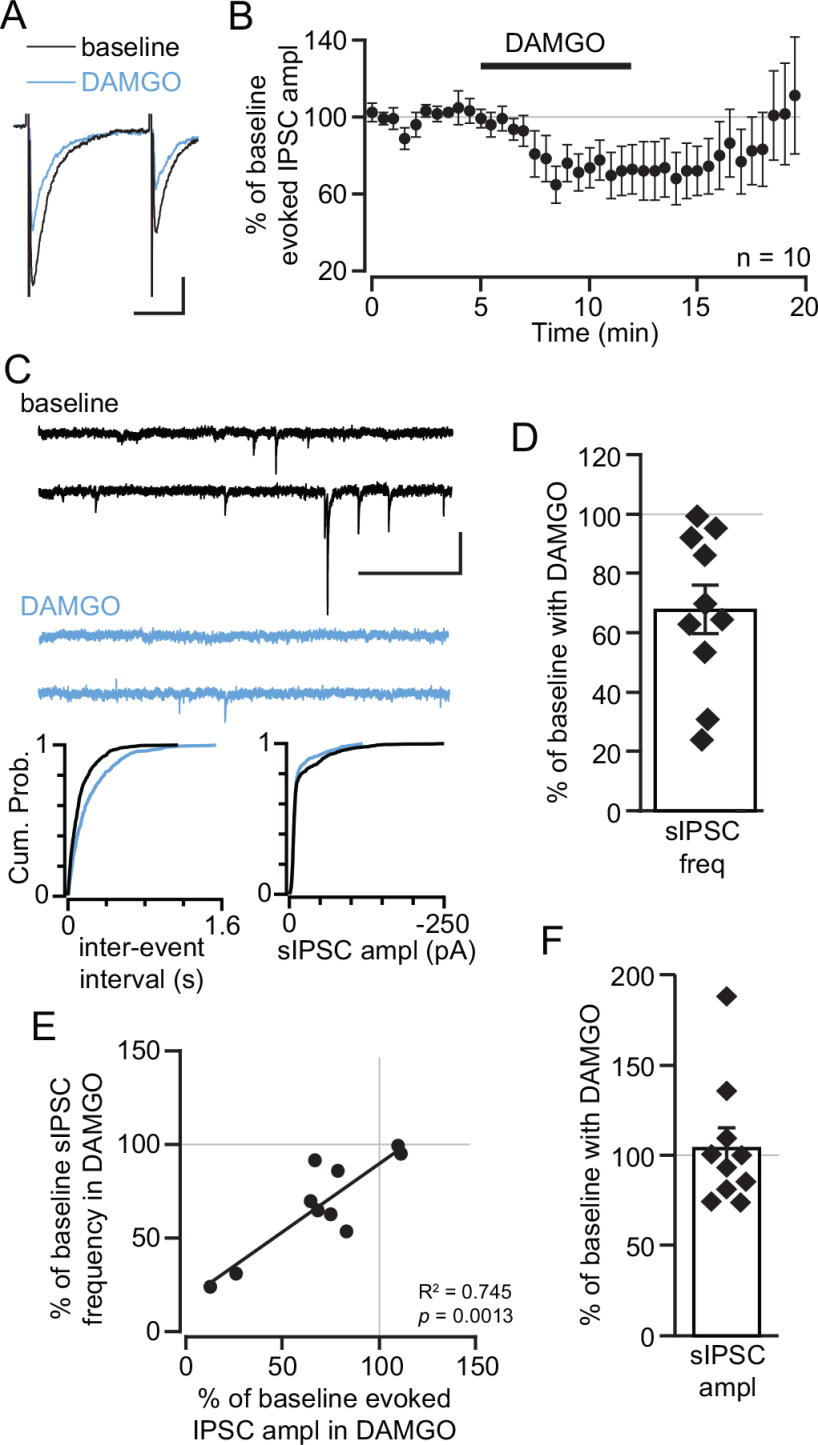
MOR inhibits GABAergic terminals onto LHb neurons. (A) Example electrically stimulated IPSCs are inhibited by 500 nM DAMGO application. Scale bars 50 pA and 20 ms. (B) Time course average of DAMGO effect across all neurons tested. (C, top) Example traces of sIPSCs during baseline and in DAMGO. Scale bars 100 pA and 500 ms. Cumulative plots for the same example neuron show a shift to longer inter-event intervals in the presence of DAMGO (C, lower left) but no change in the sIPSC amplitude in response to DAMGO (C, lower right). (D) Distribution of inhibition of sIPSC frequency by DAMGO across all measured neurons. In neurons where both electrically evoked IPSCs and sIPSCs were measured, there was a significant positive relationship between the DAMGO induced inhibition of evoked IPSC amplitude and sIPSC frequency (E), suggesting a presynaptic MOR action. (F) There was no consistent change in sIPSC amplitude in response to DAMGO.

Inhibition of glutamate release onto LHb neurons is another mechanism by which MOR activation could decrease activity of LHb neurons. We first examined DAMGO effects on electrically evoked excitatory postsynaptic currents (EPSCs). Under these recording conditions, the non-NMDAR antagonist DNQX (10 μM) completely eliminated the EPSCs (96 ± 1% blockade, n = 4). In 3 out of 10 neurons, 500 nM DAMGO caused a significant decrease in the amplitude of the evoked EPSCs (Fig 2A and 2B). To estimate the onset time, in the 3 neurons with a significant DAMGO response we calculated the time to half maximal effect to be 2.39 ± 0.06 min after DAMGO was added to the perfusion solution. The PPR, an indication of release probability, increased in all 3 neurons that showed a change in evoked EPSC amplitude, suggesting a presynaptic decrease in the probability of release (Fig 3F). There was a significant difference in this difference in PPR before and after DAMGO between those cells in which we observed a DAMGO effect on the evoked EPSC amplitude and those in which we did not (change in PPR 0.269 ± 0.073 vs 0.005 ± 0.066, respectively, *p* = 0.048). This suggests that DAMGO has a presynaptic inhibitory effect on glutamate release. There was no difference in the baseline PPR between those neurons where DAMGO inhibited the evoked EPSC and those where it did not, suggesting no baseline difference in the probability of release across the inputs onto these two neuronal populations (0.75 ± 0.19 vs 0.65 ± 0.10, respectively; *p* = 0.6; Fig 2F). To further support the presynaptic site of MOR action, we measured the frequency and amplitude of spontaneous EPSCs (sEPSCs) in the same neurons. sEPSC frequency decreased with DAMGO application to some extent in most neurons, but this change was much larger in cells where evoked EPSC amplitude decreased, and these two measures were significantly correlated (Fig 2C–2E and 2G). Across all neurons, there was no change in sEPSC amplitude in the presence of DAMGO (94 ± 6% of baseline, n = 12; Fig 2E), however there was also some attenuation of sEPSC amplitude across the 3 neurons where DAMGO markedly inhibited the evoked EPSC (76 ± 17% of baseline, n = 3; Fig 2E). There was no relationship between the change in sEPSC amplitude in DAMGO and the evoked EPSC inhibition (Fig 2H). Together, these data are consistent with a presynaptic MOR inhibition of glutamate release onto a subset of LHb neurons.

We observed some baseline differences in sEPSC measures between those neurons where the evoked EPSC was inhibited by DAMGO and those that were insensitive. The baseline sEPSC frequency was greater in those neurons where DAMGO inhibited the evoked EPSCs (7.5 ± 2.7 Hz vs 3.3 ± 0.4 Hz, *p* = 0.047). The mean baseline amplitude of sEPSCs was also significantly larger in those neurons where DAMGO inhibited evoked glutamate release (-24 ± 2 pA vs -15 ± 2 pA, *p* = 0.039). In addition to this presynaptic MOR action, DAMGO induced outward currents in 2 of the 3 neurons where we observed inhibition of glutamatergic EPSCs; no outward currents were observed in the 7 neurons where DAMGO did not affect evoked EPSCs (*p* = 0.07; 2-tailed Fisher exact test). This arrangement is similar to MOR control of nociceptive transmission in spinal cord substantia gelatinosa neurons that are directly hyperpolarized by DAMGO and have primary afferent glutamatergic input that is presynaptically inhibited by DAMGO [1, 2].

Since we found that a subset of LHb neurons are hyperpolarized by MOR activation, and LHb neurons make local glutamatergic synapses and fire spontaneously in the slice [5, 6], it is possible that the ubiquitous decrease in sEPSC frequency we observed in response to DAMGO (Fig 2E) was partly due to MOR inhibition of other LHb glutamatergic neurons in the slice. To isolate presynaptic MOR actions at glutamate terminals we measured DAMGO effects in the presence of the Na^+^ channel blocker tetrodotoxin (TTX, 500 nM) to prevent action potential firing. On average, TTX application decreased the frequency of sEPSCs to 77 ± 16% of baseline (n = 7 neurons). TTX application did not change the event amplitude (107 ± 14% of baseline amplitude, n = 7). In the presence of TTX, 5 neurons were tested for DAMGO effects on miniature EPSCs (mEPSCs). Consistent with the evoked EPSC data, the DAMGO induced change in mEPSC frequency was significantly greater (*p* = 0.005) in the 2 neurons where DAMGO caused an outward current (64 ± 7% decrease in frequency from baseline) compared to the 3 neurons with no DAMGO induced outward current (9 ± 4% decrease in frequency from baseline). There was a trend towards a decrease in mEPSC amplitude across all 5 neurons tested with DAMGO (baseline: -23.4 ± 4.3 pA vs DAMGO: -13.8 ± 1.9 pA; *p* = 0.08), and mEPSC amplitude decreased in both of the neurons where DAMGO caused a decrease in mEPSC frequency (56 ± 4% of baseline). Such a decrease in mEPSC amplitude could either be caused by a postsynaptic decrease in AMPAR function or due to selective MOR inhibition of terminals producing larger mEPSCs. The fact that mEPSC frequency is decreased only in a subset of LHb neurons when firing of other neurons in the slice is blocked, compared to the more uniform inhibition of sEPSC frequency, indicates that a significant number of MOR sensitive LHb neurons make local glutamatergic synapses onto other LHb neurons that are DAMGO-insensitive.

We also tested whether MOR activation affected GABAergic synapses in the LHb. We confirmed that the evoked potentials recorded in these experiments were GABA_A_R mediated: 20 μM gabazine eliminated the evoked potentials (97 ± 6% inhibition, n = 4). In most LHb neurons (9/11) we observed at least some inhibition of evoked GABA_A_R mediated IPSCs (29 ± 9% inhibition, n = 11; Fig 3A and 3B). To estimate the onset time in the 9 neurons with a significant DAMGO effect we calculated the time to half maximal effect to be 2.25 ± 0.35 min after DAMGO was added to the perfusion solution. This is similar to the onset timing of the inhibition of evoked EPSCs (*p* = 0.8 ANOVA). DAMGO also inhibited the sIPSC frequency in LHb neurons (Fig 3C and 3D). The inhibition of evoked IPSCs correlated with the inhibition of sIPSC frequency in the same neurons (Fig 3E). There was no observed change in sIPSC amplitude (104 ± 11% of baseline, n = 11; Fig 3F), consistent with a presynaptic MOR effect. We also found a small but significant increase in the PPR in response to DAMGO across all 11 neurons (baseline = 0.74 ± 0.10, DAMGO = 0.82 ± 0.18, *p* = 0.01 paired 2-tailed Student’s t-test). Together, these data demonstrate a significant presynaptic MOR inhibition of GABA release onto LHb neurons.

## Discussion

Here we show that the MOR selective agonist DAMGO inhibits a subset of LHb neurons by two distinct synaptic mechanisms: postsynaptic hyperpolarization and presynaptic inhibition of glutamate release. Our evidence indicates that both actions are largely restricted to a single, distinct subset of LHb neurons. If increased activity in this subset of LHb neurons contributes to pain or an aversive state, these inhibitory actions could contribute to MOR induced analgesia or negative reinforcement.

**Fig 4:**
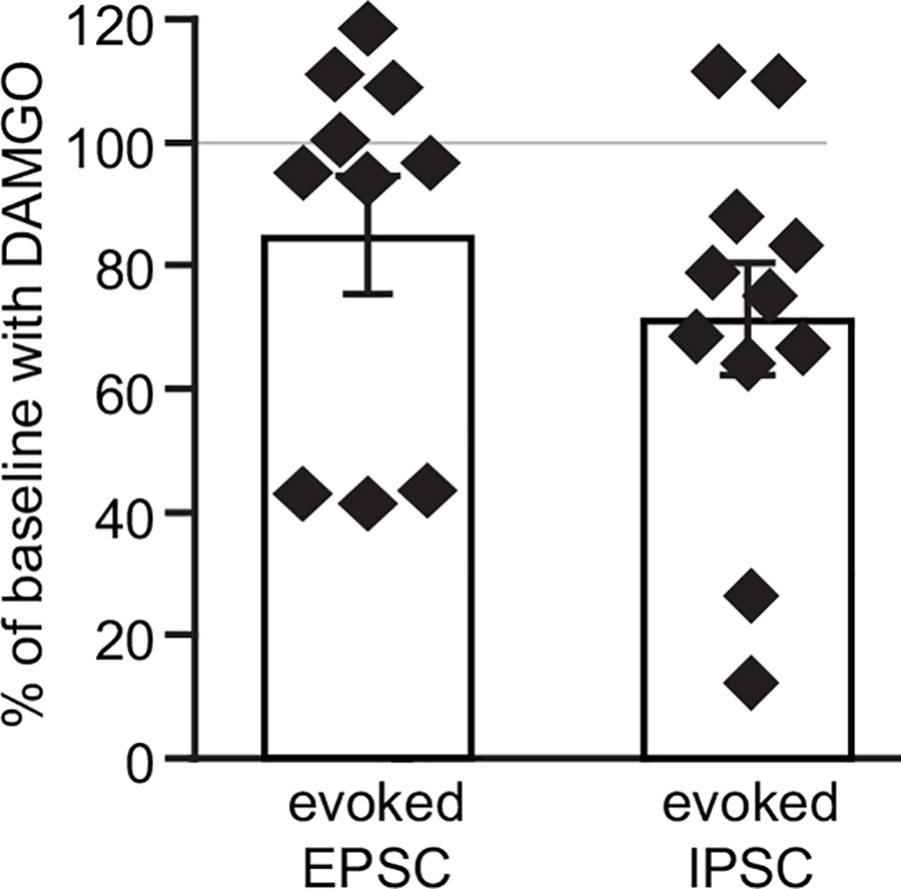
The distributions of MOR inhibitions of release varies between glutamate terminals and GABA terminals. The observations of cells tested for DAMGO effects in evoked EPSCs appears bimodal, with one cluster near 40% of baseline and another apparently insensitive, near 100% of baseline. In observations of DAMGO effects on GABA_A_R mediated IPSCs, most responses were clustered around 75% of baseline, and only 2 cells clearly showed no response to DAMGO.

We also found presynaptic inhibition of GABA release onto a larger subpopulation of LHb neurons (Fig 4). Given that the proportion of neurons in which we observe this effect is significantly larger than those with an inhibition of glutamate release (2-tailed Fisher exact test, *p* = 0.03) it is likely that there are LHb neurons which receive GABA inputs that are inhibited by MOR while their glutamate inputs are unaffected. The predominance of inhibition of GABA release would most likely have a net excitatory effect on these LHb neurons; however, the behavioral impact of such an action is unclear. In this regard, it is critical to determine whether this presynaptic inhibition of GABA release is more consistent within a subset of LHb neurons, such as those with a particular projection target. For instance, the LHb efferent projections to the VTA, DRN, and RMTg arise from different LHb cell bodies [11, 13] and therefore may contribute differentially to behavior.

Even if consistent patterns of MOR synaptic actions based on efferent projection target emerge, the interpretation of the presynaptic inhibitory action will be challenging. The LHb receives inputs from a variety of sources and different inputs can selectively target LHb neurons with different downstream targets. For instance, nucleus accumbens inputs target LHb neurons that project to the DRN/periaqueductal grey [14], and the inputs from the lateral hypothalamic area synapse onto LHb neurons that project to the VTA and RMTg [24]. Even within the LHb projection to the VTA, for example, there are synapses onto both dopamine and GABA neurons [25], whose actions can be opposing [26, 27]. Similarly, subsets of LHb neurons projecting to various other brain regions including the RMTg and DRN could target several different neuronal populations with different and in some cases opposing behavioral actions. In addition, these parallel circuits could interact within the LHb through the local glutamatergic connections. In this way, MOR expression may affect the activity of a wider group of LHb outputs even though the direct somadendritic inhibition occurs in a relatively restricted subset of neurons. Furthermore, presynaptic inhibition will only be significant behaviorally if the inhibited input is active. It will be essential in future experiments to study the effects of MOR agonists on neurotransmitter release from terminals that are identified as arising from specific inputs, as well as in particular projections.

It is somewhat surprising that MOR inhibited GABA release onto more neurons than glutamate release (Fig 4), given that several major LHb inputs (e.g. from the EPN and VTA) co-release GABA and glutamate, and anatomical evidence indicates that most axon terminals in the LHb contain both neurotransmitters [28, 29]. One possibility is that these MORs are expressed on inputs that are exclusively GABAergic, potentially from sources such as the nucleus accumbens whose projection neurons are not thought to synthesize glutamate. Although there is evidence for co-release from single vesicles in LHb [29], another possibility is that even in terminals that release both GABA and glutamate, vesicles with each neurotransmitter are segregated and have different release sites, such that the MOR signaling could be restricted to GABA release sites. In fact, there is evidence that G protein coupled receptor effects can be highly compartmentalized, even within individual spines [30], as is the Ca^2+^ influx contributing to neurotransmitter release [31]. As the physiological function of co-release remains to be determined, it is too early to speculate on the role of these presynaptic MORs. Future studies will be directed at investigating which specific GABAergic inputs are modulated by MORs activation, and how this relates to GABA-glutamate co-release.

## Conclusions

The LHb has emerged as a critical node of converging circuits that participate in pain transmission and modulation. It is ideally situated as a target for MOR mediated pain control. Here we discovered three distinct synaptic actions of MOR that predominate in different subpopulations of LHb neurons; in one subset MOR activation hyperpolarizes the neuron and presynaptically inhibits glutamate release, effects consistent with an analgesic action. Presynaptic inhibition of GABA release was also observed in a larger subset of LHb neurons. Further experiments will be required to determine how these synaptic actions control pain and contribute to opioid reward.
